# Regulation of host cellular gene transcription by Bovine Leukemia Virus (BLV) Tax, wild type and mutants

**DOI:** 10.1186/1742-4690-8-S1-A18

**Published:** 2011-06-06

**Authors:** Mariluz Arainga-Ramirez, Eri Takeda, Yoko Aida

**Affiliations:** 1 Medical Genome Sciences Department, The University of Tokyo, Saitama, 351-0198, Japan; 2 Viral Infectious Diseases Unit, RIKEN, Saitama, 351-0198, Japan; 3 Viral Infectious Diseases Unit, RIKEN, Medical Genome Sciences Department, The University of Tokyo, Saitama, 351-0198, Japan

## 

BLV is associated with enzootic bovine leukosis, which is the most common neoplastic disease of cattle, and is closely related to HTLV-1. The Tax protein of BLV is a transcriptional activator of viral replication, a key contributor to the oncogenic potential and a positive and negative regulator of apoptosis. In our previous study, we identified interesting mutant BLV Tax with elevated (TaxD247G) or reduced (TaxS240P) transactivation activity on the replication and propagation of BLV. However, the effects of mutation on other functions besides its function as a transcriptional activator are unknown. To address this, by microarray analysis, we here identified different cellular genes in response to Tax wild type and mutants. We constructed expression vectors encoding Tax wild type (TaxWT), TaxD247G or TaxS240P; introduced into HeLa cells and performed a microarray analysis. Up- and down-regulation of 122, 118 and 139 genes were detected for TaxWT, TaxS240P and TaxD247G, respectively, but TaxS240P down regulated more genes than TaxWT or TaxD247G. For comparative purposes, we distributed the genes according to Gene Ontology processes and identified expressed genes involved in transcription, signal transduction, immune response, cell proliferation, cell growth and apoptosis, which were affected by Tax. Interestingly, there was a notable fold change difference between up-regulated genes involved in transcription, signal transduction and cell proliferation induced by TaxS240P or TaxD247G. In addition, genes down regulated related to immune/viral response belonged to the interferon family. Thus, our result may be useful for understanding Tax functions and its regulation which cellular factors.

